# Patients’ experiences of cognitive behavioural therapy integrated with activity pacing: a qualitative study

**DOI:** 10.1186/s12885-025-13971-x

**Published:** 2025-04-11

**Authors:** Mikiyas Amare Getu, Mirgissa Kaba, Adamu Addissie, Edom Seife, Panpan Wang, Xianbin Zhang, Changying Chen, Eva Johanna Kantelhardt

**Affiliations:** 1 Guangdong Key Laboratory for Biomedical Measurements and Ultrasound Imaging, National Regional Key Technology Engineering Laboratory for Medical Ultrasound, School of Biomedical Engineering, Shenzhen University Medical School, Shenzhen, 518060 China; 2 School of Nursing, Woldia University, Woldia, Ethiopia; 3 Global Health Working Group, Martin-Luther-University, Halle (Saale), Germany; 4 School of Public Health, College of Health Sciences, Addis Ababa University, Addis Ababa, Ethiopia; 5 Institute of Medical Epidemiology, Biostatistics and Informatics, Martin-Luther-University, Halle (Saale), Germany; 6 Department of Oncology, College of Health Science, Tikur Anbessa Specialized Hospital, Addis Ababa University, Addis Ababa, Ethiopia; 7 School of Nursing and Health, Zhengzhou University, Zhengzhou, China; 8 Department of General Surgery, Institute of Precision Diagnosis and Treatment of Digestive System Tumors, Carson International Cancer Center, Shenzhen University General Hospital, Shenzhen University, Shenzhen, China; 9 Institute of Hospital Management of Henan Province, Zhengzhou, China; 10 The First Affiliated Hospital of Zhengzhou University, Zhengzhou, China; 11 Department of Gynaecology, Martin-Luther-University, Halle (Saale), Germany

**Keywords:** Activity pacing, Breast cancer, Cognitive behavioural therapy, Qualitative

## Abstract

**Background:**

Our previous trial integrating cognitive behavioural therapy with activity pacing (CBT-AP) demonstrated the efficacy of CBT-AP in reducing cancer-related fatigue and depression, as well as improving the quality of life for patients with breast cancer. However, the range of subjective patient experiences on the effect of CBT-AP, including its content, context, and approach, has not been fully explored. This study aimed to explore patients’ experiences of CBT-AP among breast cancer patients.

**Methods:**

Patients with breast cancer who attended CBT-AP sessions were interviewed. The interviews continued until data saturation was achieved, with no new findings emerging. A total of 20 women, aged 24 to 62, at various stages of cancer and undergoing chemotherapy were involved in the study. The collected data was transcribed, translated and coded following themes developed in line with the objective of the study, ensuring sensitivity to context, rigour, transparency, and impact throughout the research process.

**Results:**

The data identified six major themes: the content of the therapy, the context of the therapy, experiences with the implementation of the therapy, benefits of the therapy, the therapeutic approach, and recommendations. Findings revealed that participants described all components of CBT-AP as important, with the content addressing common symptoms related to the disease and its treatment. Participants reported positive effects on their physical, psychological, and social health following the therapy. While the majority preferred face-to-face sessions, a significant number favoured a combination of face-to-face and telephone sessions. The participant’s manual was found to be easily understandable and clear.

**Conclusion:**

The findings suggest that participants had positive experiences with the therapy. Based on these experiences, it is recommended that specific components and delivery methods of CBT-AP, such as patient-centered content and flexible delivery options, be considered key factors to enhance its acceptability and feasibility. While this study highlights CBT-AP’s potential to improve quality of life of breast cancer patients, further research is needed to evaluate its broader application and long-term impact in various healthcare settings, particularly for diverse patient populations.

## Introduction

Breast cancer was the most commonly diagnosed cancer and the leading cause of global cancer incidence in 2020. It accounted for an estimated 2.3 million new cases, representing 11.7% of all cancer cases. Breast cancer ranked as the fifth leading cause of cancer mortality worldwide, with 685,000 deaths reported in 2020 [1]. The American Cancer Society estimated that in the United States in 2023, there would be 297,790 new cases of invasive breast cancer and approximately 43,700 deaths among women due to this disease [2].

A diagnosis of breast cancer is emotionally challenging and brings with it a myriad of physical, psychological, and social implications for those affected. Most individuals diagnosed with breast cancer experience cancer-related fatigue, anxiety, depression, and insomnia both after diagnosis and during chemotherapy [3]. These symptoms are closely associated with disability and a reduction in quality of life (QoL) [4].

Research suggests that patients with disabilities, including those stemming from physical or emotional health challenges associated with illness, may experience higher levels of fatigue [5], which in turn impacts QoL [6, 7]. For example, physical limitations can reduce activity levels, leading to inactivity and increased fatigue. Emotional challenges, such as depression or anxiety, can further exacerbate fatigue, creating a cycle in which fatigue and disability reinforce each other [5, 6]. Implementing psychosocial therapy tailored to the unique needs of patients with breast cancer can address disability-related concerns and improve QoL by promoting adaptive coping strategies, resilience, and emotional well-being, thereby reducing the likelihood of maladaptive behaviours [4]. Cognitive behavioural therapy (CBT) [8] and activity pacing (AP) have emerged as promising approaches, offering potential benefits for patients with breast cancer [9]. CBT, a well-documented psychotherapeutic technique, focuses on identifying and altering dysfunctional thoughts and behaviours. It has been shown to reduce psychological distress, depression, and fatigue in patients with cancer [10, 11]. Similarly, AP, derived from energy envelope theory—an adaptive strategy that involves carefully balancing activity and rest, as well as perceived and expended energy—has proven effective in managing symptoms and improving overall QoL [9]. This approach operates at the practical level of daily activities, enabling individuals to pace themselves strategically, thereby mitigating symptoms and contributing to an improved QoL [9].

CBT and AP are distinct components of non-pharmacological interventions recommended by the NCCN guidelines for managing fatigue [12]. These approaches complement each other. Therefore, we have integrated CBT and AP interventions [13] to enhance the efficacy of CBT in reducing fatigue and improving QoL. The combined CBT-AP intervention was delivered over seven sessions: three face-to-face and four telephone sessions. The intervention aimed to reduce cancer-related fatigue and improve the QoL of patients with breast cancer undergoing chemotherapy. Details of the therapy and its outcomes were presented by Getu et al. (2023).

Our CBT-AP trial (Trial registration number: PACTR202008881026130, Registration date: 24 August 2020) demonstrated the efficacy of CBT-AP in reducing cancer-related fatigue and depression and improving the QoL of patients with breast cancer [14]. However, the range of subjective experiences encompassing the physical, psychological, and social effects among patients has not been clearly described. Additionally, our previous study did not address patients’ experiences regarding the implementation of the therapy, the content of the intervention, the patient manual, the therapeutic approach, or the qualities of the therapist. Therefore, a qualitative assessment is particularly appropriate for identifying unmeasured patient concerns about the intervention. Our objective is to provide valuable insights into the effectiveness and acceptability of the combined approach through an in-depth exploration of how patients with breast cancer perceive and navigate the combined therapeutic modality of CBT and AP. The findings of this study could inform revisions to the intervention package based on patients’ lived experiences, enhancing the acceptability and uptake of this new intervention and ultimately reducing participant dropout rates. Furthermore, this qualitative study contributes to a comprehensive understanding of the impact of the integrated therapeutic approach on patients’ physical, psychological, and social well-being [15].

This study is part of a broader project assessing the effect of the newly developed CBT-AP intervention [14]. This phenomenological study was conducted to explore the experiences of patients with breast cancer who attended all CBT-AP intervention sessions.

## Methods

### Study design

This study employed a descriptive phenomenological design.

### Participant and recruitment

#### Eligibility criteria

Women with breast cancer who had undergone chemotherapy and were offered CBT-AP were included in the study. Patients who were seriously ill, had a psychiatric illness or uncontrolled medical conditions, or who declined to participate in the study were excluded. In total, 31 patients were in the intervention group to receive CBT-AP, of whom 27 fully attended all CBT-AP sessions. We purposively selected from these 27 individuals, aiming for maximum variation in terms of age, marital status, educational level, stage of cancer, and time since diagnosis to capture a range of experiences. Participants who were willing to participate provided written consent.

#### Data collection materials and methods

All interviews were conducted in a private room at Tikur Anbessa Specialized Hospital and lasted approximately 45 to 60 min. The setting ensured that no one could oversee or overhear the interview.

Data were collected using individual semi-structured interviews to gather rich and detailed personal data and experiences regarding CBT-AP from each patient. The socio-demographic and clinical characteristics of the participants were obtained from the participants themselves and their medical records.

The interview guide focused on the participants’ responses to CBT-AP, with their answers used to generate further probing questions for clarification. The interview guide provided questions and prompts to facilitate conversations between the interviewer and participants. It was developed in accordance with the Consolidated Criteria for Reporting Qualitative Research guidelines, as shown in Table 1. Two interviewers experienced in qualitative research were trained on the interview guide, open-ended questioning, probing, and active listening to elicit rich and meaningful data and ethical procedures.

**Table 1:** Interview guide to explore the experiences of CBT-AP among patients with breast cancer

1.	What was your experience with the therapeutic approaches (telephone and face-to-face)? **Probe**: Acceptability, suitability, effectiveness, appropriateness to the content, personalisation of the intervention, and your experience with other patients in the group therapy.
2.	How did you find the content of the intervention for both telephone and face-to-face sessions? **Probe**: Introductory session about breast cancer, its symptoms and treatment, main content (CBT, AP, sleep disturbance management, dysfunctional thought management), and the closing session (goal setting and action plan).
3.	Can you describe your experience with the intervention manual and worksheet? **Probe**: Understandability, clarity, and applicability. Prompt: How easy or difficult was it to work through or implement? What difficulties did you encounter?
4.	How did you find the context of the intervention? **Probe**: Setting, timing, regularity/weekly schedule, length of each session, and the total number of sessions.
5.	How did you find the therapists during the face-to-face and telephone sessions? **Probe**: Attention, compassion, respect, caring, sympathy, timing, language, competence, and problem-solving ability.
6.	Describe the effect of CBT-AP on your life: physically (e.g., symptoms such as fatigue, pain, insomnia), psychologically (e.g., thoughts, emotions, behaviour), and socially (e.g., interaction with family, friends, community, social support).
7.	What barriers and facilitators did you experience when participating in CBT-AP? **Probe**: Content, context, approach, therapist.
8.	What suggestions do you have for improving CBT-AP? **Probe**: Content of the intervention, intervention module, worksheet, therapeutic approaches (telephone and face-to-face modes), and context of the intervention (setting, timing/weekly schedule, regularity, length of each session, total number of sessions), therapist (attention, compassion, respect, caring, sympathy, timing, language, competence, problem-solving ability).
9.	How have you incorporated CBT-AP into your day-to-day life after completing the therapy sessions? **Probe**: Intervention manual, worksheet.

We initially invited 27 participants based on the inclusion criteria, but interviews continued until data saturation was reached, and no new findings emerged after data were collected from 20 participants. This was cross-checked by two data collectors during data collection. Data saturation, guided by the principle of theoretical sufficiency, was achieved when sufficient information had been gathered to replicate the study, new participants were not adding new information, and the identified themes provided a comprehensive explanatory framework. Data saturation is related to data collection and determines whether to continue the interviews or conclude that the data are saturated [16, 17]. Failure to achieve data saturation can negatively affect the validity of the study [18]. To affirm full saturation, two additional individual interviews were conducted; however, no new information emerged, reinforcing the thoroughness of the data collection process.

All interviews were audio-recorded and transcribed verbatim. After transcription, the transcripts were translated into English for analysis by the research team (MAG and PW). If discrepancies arose, the two researchers discussed them, and if they could not reach a consensus, a third person was brought in to reconcile the differences. Transcripts were returned to the participants if discrepancies were identified for correction and/or comment.

### Data analysis

The data analysis was conducted using the six-phase thematic analysis developed by Braun and Clarke [19] in 2006. Thematic analysis was performed using MAXQDA Standard 12 [20], which helped organise relevant codes and visualise important elements through various built-in tools.

### Data management and quality assurance

All aspects of the research were conducted with sensitivity to context, rigour, transparency, and impact. Regarding sensitivity to context, word-for-word quotes and comments related to body language were considered during the analysis.

The principal investigator and the research assistant independently reviewed transcripts and coded the data using the transcription document. After independent coding, they discussed the consistency of themes and sub-themes. Sample codes were checked against transcripts by one of the authors (MK). The transcribed data were also given to two other experts in qualitative studies to define themes and sub-themes, ensuring the validity and reliability of the data. The outcomes of coding among the researchers and the invited experts were compared, and any discrepancies were resolved. Finally, themes and sub-themes that could be used for discussion were identified.

To maintain data quality and control, regular scheduled meetings were held among the research team to address any issues that might arise during the study. Audio-recorded interviews and transcripts were checked by each participant to ensure credibility. The participation of qualitative study experts in the analysis process and the provision of a detailed description of the research methods ensured dependability. Confirmability was achieved through bracketing, where notes on personal feelings, biases, and insights were recorded immediately after each interview to ensure researcher perspectives did not influence data interpretation.

### Human ethics and consent to participate

Our study was conducted in line with the principles of the Declaration of Helsinki. Approval was granted by the Institutional Review Board of Zhengzhou University (number: ZZUIRB 2020-10, Date: 18 June 2020) and Addis Ababa University (number: 101/20/Onco, Date: 28 October 2020). Informed consent was obtained from all participants for the post-intervention interview.

## Results

### Participants’ characteristics

In total, 20 women with breast cancer aged 24 to 62 years who participated in the CBT-AP intervention were included in this study. Among these participants, 8 had stage II breast cancer and 20 completed all intervention sessions. Additionally, 9 of the 20 participants were divorced. The characteristics of the participants are shown in Table 2.

**Table 2:** Socio-demographic characteristics (*n* = 20)

Participants	Age (years)	Marital status	Education level	Stage of cancer	Time since diagnosis
P01	41	Married	Secondary	II	2 years
P02	53	Divorced	Secondary	II	1 year 9 months
P03	42	Married	Secondary	III	1 year 7 months
P04	40	Divorced	PhD candidate	I	1 year 9 months
P05	32	Divorced	Secondary	III	1 year 7 months
P06	32	Single	Secondary	III	1 year 4 months
P07	62	Divorced	Primary	III	1 year 3 months
P08	24	Single	College	II	1 year 7 months
P09	39	Single	College	III	4 years
P10	30	Married	Diploma	II	2 years
P11	30	Divorced	Secondary	IV	3 years
P12	33	Divorced	Secondary	IV	2 years 9 months
P13	42	Married	Secondary	II	1 year 6 months
P14	44	Divorced	Secondary	II	1 year 7 months
P15	34	Single	Primary	II	1 year 6 months
P16	35	Divorced	Secondary	I	1 year 3 months
P17	50	Married	Secondary	III	1 year 3 months
P18	41	Divorced	Secondary	I	1 year 4 months
P19	31	Single	Secondary	III	3 years
P20	52	Single	Primary	II	1 year 7 months

### Overview of themes

The analysis of the qualitative data led to the identification of six major themes: the content of the therapy, the context of the therapy, experiences with the implementation of the therapy, the benefits of the therapy, the therapeutic approach, and recommendations. The structure of the themes and sub-themes are illustrated in Table 3.

**Table 3:** Thematic structure of participants’ experiences with CBT-AP

**Theme 1: Content of the therapy (CBT-AP)**
Sub-theme 1: Components of the therapy
Sub-theme 2: Content improvement
**Theme 2: Context of the therapy**
Sub-theme 1: Number of sessions
Sub-theme 2: Starting time and duration
Sub-theme 3: Place of delivery
**Theme 3: Experience with implementation of the therapy**
Sub-theme 1: General experiences about the therapy (content, approach and benefit)
Sub-theme 2: Implementation of the therapy
Adherence to the therapy after completion of session
Implications for others
**Theme 4: Benefit of the therapy**
Sub-theme 1: Physical health
Sub-theme 2: Psychological health
Sub-theme 3: Social health
**Theme 5: Therapeutic approach**
Sub-theme 1: Qualities of the therapist
Punctuality
Compassionate, respectful, and caring
Problem-solving ability
Sub-theme 2: Preferred therapeutic approach
Face-to-face
Telephone
Face-to-face and telephone
Sub-theme 3: Participant’s manual
Understandability and clarity
Applicability
**Theme 6: Recommendations**
Sub-theme 1: Availability, accessibility, and sustainability of therapy
Sub-theme 2: Optimisation of the therapy
Therapist
Context of therapy
Content and approach

### Content of the therapy

This theme describes participants’ experiences regarding the detailed content of CBT-AP and areas for improvement. It highlights the structured nature of the therapy and its importance. Additionally, it reveals the systematic approach of CBT-AP, where each session was designed to build upon the previous one, progressively addressing the patients’ cancer-related symptoms. This theme reflects how participants perceived and internalised these components, with some finding certain techniques particularly beneficial and others identifying areas that could be improved.

Participants described the therapy sessions as highly structured, with each session introducing specific techniques aimed at managing symptoms. Cognitive restructuring was a recurrent focus, where individuals learned to identify and challenge their irrational thoughts.

#### Components of the therapy

The components of the therapy included an introduction to breast cancer, chemotherapy, and its related signs and symptoms. It also encompassed goal setting, CBT-AP, sleep disturbance management, dysfunctional thought management, cognitive restructuring, coping mechanisms, and strategies for achieving goals.

Eighteen of the 20 participants found all components of CBT-AP to be very helpful. The content effectively addressed common symptoms related to the disease and its treatment.

Participants consistently emphasized the effectiveness of specific components of the therapy, particularly breast cancer education, relaxation techniques, cognitive restructuring, and social support. These components were frequently noted for providing guidance and support which improved participants’ capability and attitude in managing their health.

*The content of the therapy was very helpful*,* especially*,* section covering the introduction to breast cancer*,* its treatment*,* side effects*,* activity pacing*,* relaxation techniques*,* dysfunctional thought management*,* and social support section. [P04]*

This uniform appreciation underscores the relevance of this components in addressing the main challenges faced by participants, showing an alignment between participants interest and therapy design.

#### Content improvement

Most participants reported that the content is adequate in its present form, addresses their problems effectively, and that no improvement is needed. This sentiment was nearly universal among participants, reflecting a shared perception of the therapy as comprehensive and tailored to their experiences and concerns.

*I don’t think there is something to add or remove from the content. The content by itself is enough. [P10]*

This homogeneity implies that the therapy was well-calibrated to meet participants needs, with slight variation in suggestions for improvement.

### Context of the therapy

This theme describes the broader context within which CBT-AP was delivered, including the setting, number of sessions, duration of each session, starting time, and regularity of sessions. It addresses how these contextual factors influenced the process of therapy’s effectiveness and participant engagement. The context of the therapy highlights participants’ experiences of the therapeutic environment. Participants expressed satisfaction with the setting where therapy took place. The sessions were conducted in a calm, private setting that fostered a sense of security, allowing participants to open up about their feelings.

#### Number of sessions

Half of the participants mentioned the number of sessions for the therapy was adequate, describing it as neither too lengthy nor too brief for the scope of the content.

*For me*,* the number of sessions are not too short or too long. It is adequate for the content. [P06]*

*Regarding the number of sessions*,* seven sessions is enough. Delivering the therapy for more than seven sessions may not be helpful. [P07]*

These comments indicates that the therapy was well-suited to their capacity to engage, avoiding overloaded or insufficient sessions.

#### Starting time and duration

The therapy was delivered via face-to-face and telephone sessions. This sub-theme assessed the starting time and duration of sessions.

Most participants (70%) reported that the duration of both face-to-face and telephone sessions was adequate for the content. Most participants appreciated the regularity of the sessions. Some participants, particularly those coming from distant locations, suggested revising the start time of the face-to-face sessions to the morning.

*The duration for each session was adequate to the content and it was not boring. The morning session was suitable…The symptoms are more tolerable in the morning than afternoon. I like the regularity of the session because; I sometimes forget to remember the schedules. So*,* its regularity helped me to remember the next session easily. [P01]*

This highlights the role of timing on participants’ engagement, particularly for those who find it easier to manage fatigue or symptoms more effectively in the mornings.

#### Place of delivery

This sub-theme refers to the place where the therapy was delivered.

All participants appreciated that the therapy was delivered in a quite and private place which significantly enhanced their comfort and willingness to participate. The setting allowed the participants to feel their privacy was respected, which they believed was critical in creating conducive therapeutic environment.

*The setting for the intervention was good because it was provided in the hospital. It is a preferable place. It was given in a quiet place*,* and they kept our privacy. I am very happy about that. [P15]*

### Experience with implementation of the therapy

This theme describes the general experiences on the therapy and its implementation, encompassing sharing of one’s experiences during the therapy with other participants and continuity of practising the therapy after its completion. The implementation theme reveals the practicalities of delivering CBT-AP and how it was received by participants, noting any difficulties in adhering to the protocol, such as scheduling conflicts, the intensity of sessions, or barriers to remote delivery. Participants expressed gratitude for the therapy, highlighting its beneficial effects on their psychosocial health and coping mechanisms. For instance, one participant mentioned a shift from self-hatred to self-acceptance post-therapy, while another noted a newfound understanding that cancer is treatable rather than a death sentence. The implementation of CBT-AP techniques post-intervention varied among participants, with the majority demonstrating sustained adherence and a minority facing challenges in maintaining the learned strategies. This sustained implementation of CBT-AP was associated with positive changes in behaviour and emotional regulation, indicating the effectiveness of the therapy in promoting long-term coping. The implications of the therapy extended beyond individual benefits; participants actively shared their experiences and knowledge with others, contributing to a supportive network of individuals navigating similar challenges.

#### General experiences with the therapy

All participants reported that their general experience with the therapy were positive, reflecting a strong appreciation for support it provided. Several participants described the therapy as a unique opportunity, especially given its accessibility without payment, which they found it meaningful.

It was a good opportunity for Us. [P02]

Even though it isn’t easy to get this therapy with payment. [P18]

*Previously*,* I hated myself*,* but now thanks to God*,* I am well. I have accepted the disease after the therapy. It was beneficial. [P11]*

*I thought cancer was not curable and our fate was death*,* but after the therapy*,* I have learned it is treatable. I am feeling good about the psychotherapy. [P16]*

*The therapy was exciting. Previously*,* I conflicted with myself. After I received the therapy*,* I was very normal… I do activities and engage with the environment as an ordinary person does. If I did not follow (missed) the sessions*,* I would not have to get into myself. I want to say thank you for helping me get myself back. [cry] [P08]*.

This general experience of positive attitude towards the therapy indicates its role in modifying participant’s emotional and psychological outlook.

#### Implementation of the therapy

The implementation aspect was explored in terms of adherence to the therapy to sustainably implement CBT-AP after completion of all sessions and its implications for others, such as sharing materials and experiences.

#### Adherence to the therapy

Thirteen of the 20 participants described the sustainable implementation of CBT-AP after completing all sessions. These participants actively engaged with the therapy’s principle, using participant’s manual, completing worksheet, integrating techniques into daily routines. For instance, one participant described managing sleep disturbances, reducing anger, and practicing deep breathing during stressful moments.

*I tried to manage my sleep disturbance according to the therapy. Still*,* I am practicing the therapy regularly. Previously*,* I was angry at my children*,* and now I am avoiding it by modifying my incorrect thoughts*,* emotions*,* and behaviour. I do deep breathe exercises when I face a stressful situation. I think I have implemented it. [P04]*

*I balanced the activity and rest pattern. I walk for some distance and take transport for a balanced distance. Previously*,* I tried to finish all my work in one day and rest the other day. But*,* after the therapy*,* I schedule my activity and rest daily. [P01]*

However, the remaining seven participants did not continue with CBT-AP after the intervention was completed. They explained that this was due to pain, lack of time, or forgetfulness.

*I wanted to continue with the therapy after the intervention was completed. However*,* the pain was very severe specially after chemotherapy that I couldn’t continue. [P19]*

This variation in adherence highlights the importance of addressing individual barriers to long-term implementation for sustained benefits.

#### Implications for others

Some participants reported sharing the knowledge and experience gained from the therapy with other patients voluntarily. They also lent them the CBT-AP participant’s manual. Other patients benefited from reading the manual and experience sharing. This mutual support extended beyond individual therapy sessions, contributing to a larger, informal support network.

One participant recounted supporting another breast cancer patient who was facing emotional distress:

*I taught other patients as the therapist taught me. One of the patients had surgery*,* and both of her breasts were removed. Everything was dark for her. Unlike me*,* she doesn’t have a child. So*,* I supported her a lot according to the knowledge and experience I gained from the therapy. I recommended her to join a breast cancer support group. I lend my book [CBT-AP participant’s manual] to other patients. [P03]*

Another participant shared similar experiences:

*In addition to the benefits*,* I got to myself*,* I have also helped other stressed patients after I took this therapy. I gave the book for 3 to 4 patients to help them as it helped me. Most patients heard the side effects of chemotherapy and chose not to undergo chemotherapy. I taught other patients with dysfunctional thoughts about the disease and who decided to discontinue the treatment that a cure is possible. The therapy helped not only me but also many other patients. Many patients thanked the producers of the therapy. [P01]*

These responses suggest that the therapy’s positive impact extended beyond the individual benefits, fostering a supportive community and encouraging others to maintain treatment adherence and a hopeful outlook.

### Experienced benefit of the therapy

This theme focuses on the outcomes and participant’s perceived benefits of CBT-AP. This encompasses physical, psychological, and social health improvements reported by the participants after receiving CBT-AP.

Findings revealed that CBT-AP reduces fatigue, anxiety, and depression. Furthermore, participants pointed out its long-term benefits including improved resilience and confidence in managing psychosocial problems. This theme considers both immediate and sustained effects, noting any variations in benefits among different participants. Participants described feeling more in control of their thoughts and emotions, with cognitive restructuring techniques proving especially helpful in reducing irrational fears.

*I had a dysfunctional thought about the disease. I thought everyone with cancer would die immediately after diagnosis. Therefore*,* I was stressed. However*,* following the therapy [CBT-AP]*,* my dysfunctional thought was corrected. [P02]*

The benefits of CBT-AP extended beyond the therapy sessions, with participants reporting lasting improvements in their overall well-being and ability to cope with stressful situations. However, the magnitude of these benefits varied, with some participants experiencing more profound changes than others.

#### Physical health

The therapy resulted in improved physical health such as reduced fatigue and muscle tension, which enabled participants to resume daily activities.

*My physical condition has improved. I can cook my children food and accompany them to school. My fatigue is reduced. I forget the illness and I don’t remember it. [P18]*

#### Psychological health

Seventeen of the 20 participants expressed a positive impact of CBT-AP on their psychological health. Many participants shared insights on how the therapy influenced the management of dysfunctional thoughts, sleep disturbances, fear, and anxiety.

*The impact of the therapy surpasses my ability to articulate. I have experienced significant improvement. Previously*,* I had so many lousy feelings. Now*,* I have almost forgotten that I have the disease. When I remember that time (symptomatic period) and my present condition… [Laugh]…very [Laugh] different. It had better improvement on my psychological well-being. [P02]*

Four participants explained that the therapy helped them to build their confidence and motivation.

*My attitude towards the disease was terrible before the therapy [CBT-AP]. I thought the chemotherapy would kill us. After I joined the therapy [CBT-AP]*,* I became strong and confident.*

Three participants said they became hopeful, their everyday fear and stress about the disease reduced, and they even forgot about the disease after the therapy.

*Regarding psychological health*,* I have learned how to modify dysfunctional thoughts. The disease results in anxiety*,* depression*,* and negative thoughts. Previously*,* I was hopeless and decided to discontinue the treatment*,* but the therapist [CBT-AP therapists] encouraged me to continue. I have also learned about others’ experiences. [P04]*

#### Social health

The majority (65%) of the participants mentioned improved social participation and the avoidance of fear, isolation, and loneliness due to changes in physical and cosmetic appearance. The participants said the therapy built their confidence and social network. Eight of them were in the early stages of cancer.

*The physical consequences of the treatment led us to isolate ourselves from others. The therapy helps us be courageous*,* explain the disease*,* and socialise with the community. [P4]*

Nine of the 20 participants were divorced. Although marital status was not a primary theme, participants’ descriptions of their social experiences were influenced by the availability of support systems, which sometimes related to their relationship status. For instance, some participants faced challenges to participate in social activities which they associated with limited social support,.

*Honestly speaking*,* my social participation is quite limited. I often feel lonely*,* so I tend to isolate myself. As a single person with limited social support*,* I rarely attend events like weddings*,* birthdays*,* or other gatherings. [P09]*

*I was actively engaged in social activities and had good relationships with my friends and neighbors*,* but after my divorce*,* I chose to keep more to myself. [P16]*

One participant shared how the therapy helped to overcome social isolation:

*It has also a good effect on social health. Previously*,* I closed my door to others and stayed alone at my home. I refused to join others in different social gatherings because; I didn’t answer others’ questions about my hair loss. But*,* after I entered the therapy*,* I saw other patients dressing*,* courage*,* and how they act*,*…so I realised that… [cry]. It helped me cope with the situation and improve my social interaction. [P1]*

These insights underscore that CBT-AP provides broad benefits across physical, psychological, and social dimensions, contributing to overall participant resilience and well-being.

### Therapeutic approach

This theme explores the qualities of the therapist, the appropriateness of the therapeutic approach to the content, the preferred therapeutic approach, and the participants’ manual. It discusses how the approach was perceived by participants and its alignment with their expectations and needs.

The therapeutic approach theme delves into the therapeutic process, focusing on the relationship between the therapist and participants, the suitability of the delivery methods (face-to-face and telephone), and the adaptability of the therapy to individual needs. It reflects on how the approach facilitated or hindered the therapeutic process, with feedback from participants.

The therapist’s approach was a key factor in the success of CBT-AP, with participants appreciating the balance between guidance and collaboration. The therapist’s qualities in caring for and respecting the participants, as well as their ability to adapt the therapy to individual needs, were particularly noted, with personalised adjustments making the therapy more accessible and effective. However, some participants expressed a desire for more autonomy in the process, suggesting that a slightly less directive approach could have enhanced their engagement. Overall, the therapy approach was well-received, fostering a supportive environment that encouraged active participation.

#### Qualities of the therapist

The qualities of the therapist included punctuality, compassion, respect, caring, and problem-solving ability. Many participants described feeling supported, valued, and respected, which facilitated a sense of trust in the therapeutic relationship. This supportiveness encouraged active participation, contributing to participants’ perceptions of success in their sessions.

#### Punctuality

All participants reported that the therapist was punctual and attentive.

*The therapists were punctual. They arrived earlier than us. The therapists were good to me. [P10]*

#### Compassionate, respectful, and caring

All participants reported that the therapist delivered the therapy in a compassionate, respectful, and caring manner. The participants felt they were not alone and were very happy with the therapist’s approach, including their welcoming facial expressions.

*The therapists showed us a good thing. Their [therapist] treatment*,* caring*,* and their facial expression affected me more positively than the medication. They were very nice to me. [P3]*

*I remembered the young girl in her 20s who was initially very hopeless with a broken heart*,* and crying. However*,* after she completed the whole session*,* she was delighted*,* and her health condition had improved. This excellent outcome is a result of the therapist’s treatment. [P9]*

#### Problem-solving ability

Participants reported the therapist’s competence in solving their personal problems, indicating that participants valued a therapist who could tailor solutions to their unique situations.

The therapists were very good. They have good competence and problem-solving ability. [P7]

#### Preferred therapeutic approach

The therapeutic approaches included face-to-face and telephone sessions. This theme explored participants’ preferences for the therapeutic approach.

#### Face-to-face

Ten participants preferred the face-to-face approach because they could meet other participants, discuss the disease, and share experiences. Seven of these participants were single or divorced, while three were married. Additionally, participants mentioned that the face-to-face approach gave them a reason to go out and socialise.

*Both approaches are very important. However*,* I think the face-to-face approach is better for discussing our problems by meeting with other patients. It allows sharing experience with each other. [P8]*

#### Telephone

Three participants preferred the telephone approach, which allowed them to talk freely with the therapist privately without limitations. They also mentioned that this approach was preferable for those who cannot attend face-to-face sessions due to workload, personal characteristics such as shyness, or other reasons.

*The problem is with my workload for my children schooling and the therapy schedule. I prefer the therapy to be delivered via telephone because*,* I am afraid to talk. [P14]*

#### Face-to-face and telephone

Seven participants preferred a combination of face-to-face and telephone approaches. They mentioned the reasons cited above for both approaches. They emphasized the social engagement and support gained from face-to-face interactions and the convenience and privacy of telephone sessions.

*The face-to-face discussion is critical because it helps us share our experiences. It can also be a reason to go out*,* and I got the experiences of other patients with serious health problems. Regarding the telephone session*,* it allows us to ask questions privately. Both methods of delivery are recommended. Face-to-Face is better; many people have responsibilities at home and work*,* so it is essential to deliver the therapy via telephone in addition to face-to-face. [P19]*

#### Participant’s manual

A CBT-AP manual, prepared in Amharic (the national language), was distributed to all participants. The manual’s understandability, clarity, and applicability were explored.

#### Understandability and clarity

Eighteen participants reported that the manual was easily understandable and clear. The education level of 13 of these 18 participants was grade 10 or above. The manual was well-received overall, with participants reporting that it offered valuable guidance and reinforced the therapy content.

*The book preparation was good*,* described clearly. The main thing is convincing your mind. If you convince your mind*,* you can do everything. [P08]*

However, one participant aged 62 with a primary school education, mentioned that some of the parts of the worksheet sections were difficult to understand. This feedback highlights how demographic factors such as age and education level may affect individual comprehension, though the majority found the manual straightforward and clear.

#### Applicability

Participants mentioned the manual was applicable and complemented their therapy experience.

### Recommendations

This theme captures the suggestions and feedback provided by participants regarding the therapy, including proposed improvements, aspects they found most valuable, and advice for future implementations of CBT-AP. It encompasses both specific recommendations for enhancing the therapy and broader reflections on how the programme could better meet the needs of similar populations. This theme includes availability, accessibility, and sustainability of the therapy and optimisation of the therapy.

The recommendation theme offers valuable insights into how participants perceived the therapy and what changes they believe could optimise its effectiveness. Participants suggested adjustments in session frequency and length, improving accessibility to the therapy, training physicians, and the potential for follow-up sessions to reinforce learning. This theme also reflects participants’ appreciation for certain aspects of the therapy, such as the supportive group dynamics and the practical nature of the techniques taught, which they felt should be emphasised in future iterations. These recommendations underscore participants’ desire for a more adaptable and sustained approach to managing symptoms.

#### Availability, accessibility, and sustainability of the therapy

Most participants recommended making the therapy and its participant manual/book available and accessible to other patients. Their recommendations for wider availability reveal a profound sense of purpose in their recovery, reflecting a desire to share a transformative experience that they felt could ease the suffering of others.

*We have suffered a lot*,* but we have recovered now. As we have recovered and benefited from this therapy*,* I wish other patients to get this opportunity. [P01]*

One participant recommended making the therapy available for patients undergoing treatment in private hospitals. This indicates a sense of inequity, particularly from those who felt that access to the therapy was limited to certain healthcare settings, excluding others. This sense of exclusion speaks to the participants’ need for the program to be accessible beyond the public sector and available to all who need it, as this would validate the shared experiences across healthcare environments.

*Some patients from private hospitals asked me why the therapy is only for patients at governmental hospitals. Why is it not given to us in the private hospital? Why are they caring about you only? [P13]*

Two participants recommended making the therapy available on television, the internet, and other media outlets to improve its accessibility.

*It would be nice if the therapy is broadcasted on TV programs and other media alternatives. [P20]*

Most participants recommended training physicians and other healthcare providers in this therapy to improve its availability and accessibility. Their calls for more trained healthcare providers reflect their wish for compassionate, informed guidance that could reduce psychological distress and empower them as they navigate cancer treatments.

*I would recommend providing training about this therapy [CBT-AP] for the physicians to provide us a compassionate*,* respectful and caring. Most patients have no idea about the disease and its treatment*,* even though they don’t know what kind of surgery is being done. They don’t tell us about chemotherapy and its side effects. Imagine what could happen to the patient losing hair without being informed earlier. It is scary for most of the patients because of a lack of information. [P04]*

Half of the participants recommended continuing the therapy to ensure its sustainability in practising good behaviour. The completion of therapy often left some participants feeling the loss of a valued support system, revealing how deeply they had integrated CBT-AP techniques into their lives and routines.

*It would have been good if it was continued [feeling low because of its completion] [P03].*

#### Optimisation of the therapy

Optimisation of the therapy was recommended with respect to the therapeutic content and approach, context, and therapist to meet their evolving needs and enhance their understanding of the therapy’s impact.

#### Content and approach

One participant recommended recording a telephone session and listening to it at another time. Another participant suggested adding nutritional therapy to the content. These participants felt that addressing nutritional health along with emotional resilience would provide a more comprehensive approach to managing their well-being.

*Many people may not record the telephone session. For example*,* I record it and listen to it repeatedly. So*,* I think you can suggest the patients to record and listen to it in the other days. [P10]*

#### Context of the therapy

##### Length of each session

Five participants reported that the duration of the face-to-face sessions should be increased. One participant recommended giving more time for the first three sessions because they need more time to process and absorb the broad and complex content. They expressed the desire for an extended experience.

*The first three chapters are broad. So*,* I suggest giving more time for these chapters. [P04]*

##### Time of delivery

The timing of therapy in relation to other treatments, such as chemotherapy, was a significant theme, as some felt that the therapy would have provided greater support if it was given earlier in their treatment process. Their experiences reveal a sense of vulnerability and unpreparedness, which they felt could have been alleviated had CBT-AP been offered sooner.

*I recommend the therapy [CBT-AP] be offered before chemotherapy. For example*,* I did not know there was chemotherapy after surgery*,* and even after chemotherapy*,* I had no information about other treatments. So*,* there might be some patients who are like me. So*,* it is good to deliver the therapy before chemo. [P08]*

##### Number of sessions

Six participants recommended that the number of sessions should be increased.

##### Therapist

One participant reported that the therapist should encourage silent participants to express their ideas and use examples for easy understanding. This feedback reflects their need for validation and shared understanding within the therapy setting.

*The therapist shall encourage the patients to express their ideas and explain the therapy using examples for clear understanding. [P07]*

## Discussion

This study aimed to explore the experiences of participants with breast cancer who underwent CBT-AP—a newly developed psychosocial and activity-based intervention. The findings revealed that participants generally had positive experiences with the therapy, which had a beneficial impact on their physical, psychological, and social health. These results align with our previous quantitative study that demonstrated CBT-AP’s effectiveness in reducing fatigue and depression while improving overall QoL [14]. The comprehensive content of CBT-AP and its tailored approach, which was adapted to meet the needs of individual participants, likely contributed to these positive outcomes, underscoring the importance of a personalised approach in psychosocial interventions. Previous literature has also highlighted the effectiveness of CBT [21] and AP [22] in reducing symptoms, which in turn significantly improves QoL. This suggests that integrating CBT with AP may be an effective strategy for managing the multifaceted challenges faced by patients with breast cancer. Another contributing factor to the positive effects of CBT-AP may be that 95% of participants successfully completed all therapy sessions [14].

A majority of participants reported increased social participation, though some exhibited limited involvement in social activities. This was particularly evident among single or divorced participants, which may contribute to reduced social engagement, as suggested by previous research indicating that marital status can influence social support and well-being [23–25]. It has also been reported that married patients with breast cancer experience fewer psychosocial problems, likely because they can share the burden of negative emotions and receive psychological support from their spouses, which helps maintain their social participation [26, 27]. The descriptive approach allowed for an exploratory understanding of how such personal factors might intersect with patients’ psychosocial experiences [28]. In this study, participants’ feedback about limited social engagement—sometimes associated with their marital status—highlighted the need for targeted social support interventions that address the varying circumstances of both married and unmarried participants. Participants in the early stages of cancer demonstrated improved social functioning, such as interacting with friends, family, co-workers, and other patients, by overcoming fear, isolation, and loneliness following CBT-AP.

Regarding the sustainable implementation of therapy after its completion, the majority of participants reported integrating CBT-AP into their daily lives. Another study supports the beneficial effect of sustaining CBT to reduce fatigue and enhance the long-term effectiveness of CBT-AP [29]. In this study, over half of the participants were in the early stages of cancer, which may have contributed to their continued implementation of the therapy after the sessions ended. However, participants in advanced stages of cancer did not continue with CBT-AP post-intervention, possibly due to symptom exacerbation from aggressive treatment and disease progression, which made implementation challenging. This finding highlights the need for ongoing support and possibly additional interventions tailored to the needs of patients with advanced disease.

The content of the therapy was found to be very helpful, addressing common disease and treatment-related side effects identified in our previous study [7]. This comprehensiveness likely contributed to its effectiveness, as the therapy content addressed various aspects of health, including physical, psychological, and social health.

A significant aspect of the therapy was the delivery method, with many participants expressing a preference for a face-to-face approach. This preference stemmed from the opportunity to meet with other patients, discuss the disease, and share experiences. The social interactions provided by these sessions were particularly valuable for participants who felt socially isolated or had limited social engagement in their daily lives, as they provided a structured setting for emotional support and companionship. The second-largest group of participants preferred a combination of face-to-face and telephone approaches, emphasising the importance of group therapy during face-to-face sessions for emotional support and individual therapy during telephone sessions for privacy [30]. The participant’s manual was deemed easily understandable and clear, considering the average cognitive capacity of the participants, and it was translated into Amharic, the local language, with the majority of participants having an educational status of secondary school or above. However, one 62-year-old participant with only a primary school education reported difficulty with certain parts of the worksheet. This isolated feedback suggests that factors such as age and educational level may influence comprehension for some individuals. In future versions, simplifying the language or adding visual aids could improve accessibility for participants with lower educational levels or older age.

Participants also provided valuable recommendations for improving the therapy. Ensuring that CBT-AP is accessible and sustainably delivered to participants with breast cancer in hospitals is crucial, as the therapy is not yet integrated into standard cancer care. A meta-analysis of 37 controlled studies demonstrated the significance of psychosocial interventions in reducing symptoms and improving QoL in adult cancer patients [31]. Financial support for patients was another important consideration, as financial difficulties were identified as a significant barrier to accessing treatment. Our previous cross-sectional study identified financial difficulties as one of the main challenges faced by patients with breast cancer [7]. Some participants suggested increasing the total number of sessions and extending the duration of face-to-face sessions to potentially enhance the efficacy of CBT-AP. However, further study is needed to assess the effectiveness of increasing the number of sessions.

It was also recommended that CBT-AP be delivered before the beginning of chemotherapy, as patients undergoing chemotherapy experience physical and psychosocial symptoms. This timing would provide more effective insights into the disease, its treatment, side effects, and coping mechanisms.

This study has several limitations. Firstly, it only includes participants who were willing to participate, meaning that the experiences of those who declined to participate were not captured, potentially missing important perspectives. Secondly, the study focused exclusively on therapy completers, omitting the experiences of those who discontinued the therapy. These individuals may have had different views on the therapy compared to those who completed all sessions. Despite these limitations, the in-depth qualitative interviews provided valuable insights into participants’ responses to CBT-AP. Finally, the target population in this study were breast cancer patients at various stages and undergoing chemotherapy, which implies the findings may not be directly applicable to other types of cancer or patient groups. Additionally, as with most qualitative research, the generalizability of the findings is limited, and the purpose of the study was not to make broad generalizations but to gain in-depth insights into the specific experiences of participants with CBT-AP. These could inform further refinement and adaptation of the intervention to similar patient populations. Future studies should include a more diverse range of participants, including those with different types of cancer and at various stages, to provide a more comprehensive understanding of the intervention’s applicability and effectiveness.

The positive experiences reported by participants in the CBT-AP intervention underscore the potential for integrating CBT-AP within standard cancer care to enhance the overall QoL for participants with breast cancer. These findings highlight the importance of training healthcare professionals in psychosocial interventions such as CBT-AP, equipping them with the skills to support their patients’ emotional and psychological needs. By prioritising access to such therapies before and during chemotherapy, healthcare professionals can play a crucial role in mitigating common symptoms such as fatigue, anxiety, and depression, ultimately fostering a more holistic approach to cancer care that addresses both physical and psychosocial challenges. Therefore, our study provides valuable qualitative insights into the potential benefits of CBT-AP for improving quality of life among breast cancer patients. These insights provide a basis for further research and considerations within healthcare systems, which may explore broader implementation in the future with additional supportive evidence.

Future studies should explore the long-term effects of CBT-AP on cancer survivors, examining whether sustained engagement with these therapeutic strategies continues to yield psychological, social, and physical benefits over time. Moreover, expanding the scope of research to include diverse populations—including various types and stages of cancer and different demographic characteristics—could provide a more comprehensive understanding of the therapy’s adaptability and efficacy across different contexts.

## Conclusion

In this qualitative study, we explored the experiences of patients with breast cancer who underwent CBT-AP. Their experiences were summarised into six main themes and several sub-themes. The findings of this study indicate that the general experiences of the participants regarding the therapy were positive. Participants reported experiencing beneficial effects on their physical, psychological, and social health after the therapy, which suggests that CBT-AP can improve QoL. Half of the participants preferred a face-to-face approach, while more than one-fourth favoured a combination of face-to-face and telephone sessions. The number of sessions and the duration of each session were positively perceived by the participants.

Based on the experiences of the participants, it is recommended that specific components of the CBT-AP intervention, such as patient-centered content and flexible delivery options, be considered key factors to enhance its acceptability and feasibility. While the findings support the potential of CBT-AP in improving quality of life of breast cancer patients, further research is needed to evaluate its broader application and long-term impact in various healthcare settings, particularly for diverse patient populations.

## Data Availability

The data that support the findings of this study are not openly available due to reasons of sensitivity and are available from the corresponding author upon reasonable request.

## Abbreviations

AP: Activity pacing
CBT: Cognitive behavioural therapy
QoL: Quality of life
